# A new method for detecting mycoplasma in cell cultures using colocalization of DNA and membrane staining

**DOI:** 10.1016/j.bbrep.2025.102133

**Published:** 2025-07-03

**Authors:** Mengyuan Li, Shan Liu, Cheng Zhou, Mengsi Sun, Yanjie Wang

**Affiliations:** a Flow Cytometry Core Facility, Shenzhen Bay Laboratory, Guangdong, China; b Bioimaging Core Facility, Shenzhen Bay Laboratory, Guangdong, China; c Biochemistry Core Facility, Shenzhen Bay Laboratory, Guangdong, China; d Genomics Core Facility, Shenzhen Bay Laboratory, Guangdong, China

**Keywords:** Colocalization, Cytomembrane staining, DNA staining, Mycoplasma contamination

## Abstract

Mycoplasma often surreptitiously contaminates cell cultures without detection, mainly parasitizing the cell surface and thus interfering to some extent with the cells. Direct DNA staining of cell cultures often yields equivocal results and will only reliably detect heavily contaminated cultures. Interpretation is difficult in cases where degraded DNA from host cells may produce small spots of fluorescence under microscopy that mimic mycoplasma. To quickly and directly screen for mycoplasma contamination, we stained cells that were either contaminated with mycoplasma or treated with antibiotics using a combination of DNA and cell membrane fluorescent dyes. The contamination of mycoplasma could be accurately assessed by determining its colocalization with the surface of the plasma membrane. This approach minimized interference from cytoplasmic DNA components and greatly improved the accuracy of using DNA staining alone for mycoplasma detection. This study aimed to develop a colocalization method for the rapid detection of mycoplasma in culture samples, facilitating early diagnosis and treatment. It could serve as a valuable tool for the swift identification of contamination in cell cultures.

## Highlights

• Cellular DNA interferes with the results of mycoplasma elimination when using DNA staining alone.
• WGA and Hoechst co-localize on the host cell membrane to identify contamination.
• False positive results caused by cytoplasmic DNA can be effectively mitigated.

## Graphical abstract

**Figure:**
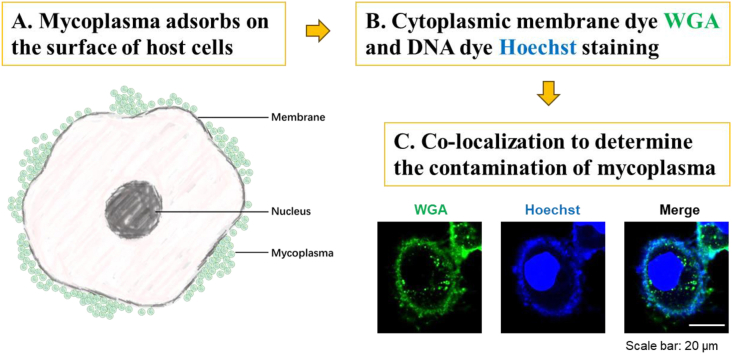

## 1 Introduction

Mycoplasma are small (300–800 nm in diameter) pleomorphic cells devoid of a cell wall that are obligate commensals or parasites with a wide range of hosts [1]. Mycoplasma contaminations are widespread in cell cultures and costly in the form of lost research [2]. Therefore, mycoplasma must be eradicated from infected cell cultures using antibiotics. Methods such as flow cytometry [3], PCR [4], and direct DNA staining [5] can be used to evaluate whether mycoplasma has been effectively eliminated. The bisbenzimidazole fluorochrome Hoechst 33342, which binds directly to DNA, is used to assess whether there are numerous points of light outside the nucleus in the background [6]. Mycoplasma-contaminated cells have brightly fluorescing nuclei, and they exhibit granular fluorescence outside the cell nucleus [5], especially on the cell membrane [7]. However, direct DNA staining can yield equivocal or even false positive results [8,9]; a possible cause is the extensive formation of micronuclei by DNA fragmentation during cell division [10]. Mycoplasma resides in a predominantly extracellular location in close association with the host cell plasma membrane [11]. This suggests that we can use the colocalization of the DNA dye, Hoechst, and the membrane dye, wheat germ agglutinin (WGA), to assess host cell contamination by mycoplasma, thus avoiding the complications arising from DNA in the cytoplasm and greatly improving the accuracy of detection.

We cultured two cell lines (B16 and MDA-MB-231) contaminated with *Mycoplasma hyorhinis*. We treated the cell cultures with anti-mycoplasma antibiotics, expecting to achieve complete eradication. We used direct DNA staining to evaluate the elimination effect of the antibiotics on the mycoplasma. The B16 cells had almost no bright spots outside the nuclei, while the MDA-MB-231 cells still had many bright spots. However, by staining the DNA and the cell membrane together, we avoided interference from the cytoplasmic DNA components and obtained consistent results with other methods.

## 2 Materials and methods

### 2.1 Cell culture

Mycoplasma-contaminated B16 and MDA-MB-231 cells were cultured in DMEM medium supplemented with 10 % fetal bovine serum in an incubator under 5 % CO_2_ at 37 °C and were treated with the anti-mycoplasma antibiotics, Myco-Off Mycoplasma Cleaner (Cat. No. D103; Vazyme, Nanjing, China), for 2 weeks.

### 2.2 Flow cytometry

Hoechst 33342 solution (Cat. No. 561908; BD Biosciences, San Jose, CA, USA) was added to cell supernatant sucked from the cell culture to achieve a final concentration of 1 μg/mL and incubated at 37 °C for 30 min in the dark. Mycoplasma cells were centrifuged at 3000g for 10 min and washed once with phosphate-buffered saline (PBS). Then, the populations and concentrations of mycoplasma in the supernatant were detected in Hoechst Blue and side scatter (SSC) channels by a flow cytometer, according to the methodology we have established [3].

### 2.3 PCR detection

A commercially available DNA extraction kit (Cat. No. 4992199; TIANGEN, Beijing, China) was used to extract genomic DNA from host cells and culture supernatants, treated with or without antibiotics. A forward primer (5′-GAACGGGATGTAGCAATACATT-3′) and a reverse primer (5′-TTTTAAGTGAAGCTGTGAAGCT-3′) were used for PCR. The PCR primers target the 16S rRNA gene sequence. The PCR conditions were as follows: 94 °C for 4 min; 35 cycles at 94 °C for 30 s, 54 °C for 30 s, 72 °C for 30 s, and 72 °C for 5 min.

### 2.4 Laser confocal microscopy

B16 and MDA-MB-231 cells were grown on confocal dishes, stained with WGA Oregon Green 488 conjugate (Cat. No. W6748; Invitrogen, Carlsbad, CA, USA) for 15 min at 37 °C and then stained with Hoechst 33342 for 15 min at 37 °C. Cells were then washed twice with 1 × PBS. Images were captured using a confocal microscope Olympus SpinSR equipped with a 60 × oil-immersion objective (NA1.3, WD 0.3 mm), and images were processed using ImageJ v.3.51. The proportion of cells with spots of the total cells in the visual field was calculated.

### 2.5 Statistical analyses

An unpaired *t*-test was used to determine whether the two groups differed significantly. All data are expressed as mean ± standard error. Statistical significance was set at *p* < 0.05, and *p*-values are indicated on the graphs. GraphPad Prism v.9.0.0 (La Jolla, CA, USA) generated all graphs.

## 3 Results and discussion

### 3.1 The detection of mycoplasma after antibiotic treatment by flow cytometry and PCR

**Fig. 1:**
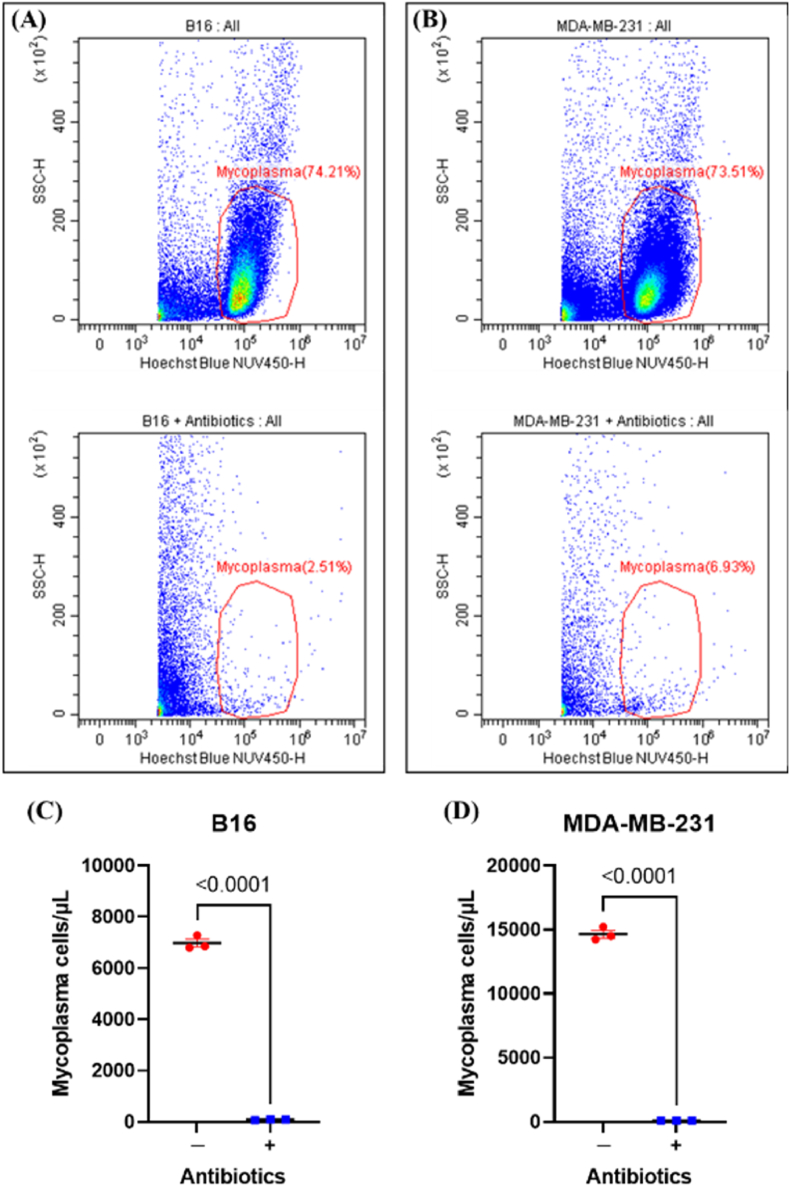
Mycoplasma concentrations (detected by flow cytometry) in the culture supernatant of two contaminated cell lines following a two-week antibiotic treatment. Hoechst Blue-H vs. SSC-H pseudo-color plots illustrated the populations of mycoplasma in the culture supernatants of contaminated B16 **(A)** and MDA-MB-231 **(B)** cells (top) and antibiotic-treated cultures (bottom). Mycoplasma concentrations (cells/μL; *n* = 3) in the culture supernatants of B16 **(C)** and MDA-MB-231 **(D)** cells (with or without antibiotic treatment), respectively.

After the B16 and MDA-MB-231 cells were treated with antibiotics for two weeks, the supernatant of the cell culture was absorbed and then stained with Hoechst. The mycoplasma in the supernatant was detected by flow cytometry (Fig. 1A and B). There were obvious mycoplasma populations in the untreated group, but the same populations disappeared in the treated group. In the supernatant of the two cell lines, the mycoplasma concentration in the antibiotic treatment group was approximately 90 cells/μL, significantly lower than that in the untreated group (Fig. 1C and D). The presence of mycoplasma in cell culture supernatants can be quantified accurately in real-time and at the single-cell level using flow cytometry. However, cell fragments and other cellular components could disperse within the plots, potentially interfering with the accurate detection of mycoplasma. This interference could lead to ambiguous results, particularly when the mycoplasma concentration was extremely low. It was advisable to use at least two different procedures for mycoplasma detection.

**Fig. 2:**
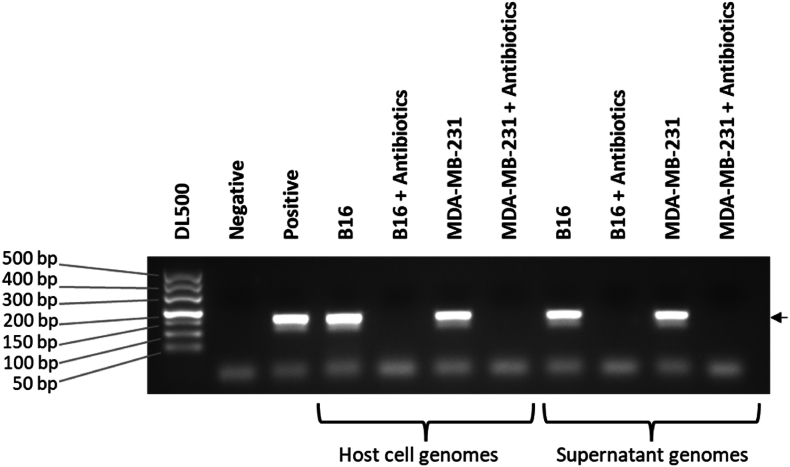
Gel electrophoresis of PCR products used to detect mycoplasma contamination in host cells and culture supernatants with or without antibiotic treatment. Specific bands at 160 bp indicate the detection of mycoplasma DNA in the samples.

Another commonly used assay, PCR, was performed to confirm the presence or absence of mycoplasma contamination. We collected the supernatant of cell cultures containing mycoplasma, and their host cells (B16 and MDA-MB-231), and extracted genomic DNA, which were used as templates for the PCR experiments. The results showed that the antibiotic-treated supernatant and host cell genome were negative for mycoplasma (Fig. 2). The PCR results were clear and incontrovertible; the host cells were cleared of mycoplasma. However, we got conflicting results when we used Hoechst direct DNA staining on the cells.

### 3.2 Direct DNA and membrane staining improved the accuracy of mycoplasma detection

**Fig. 3:**
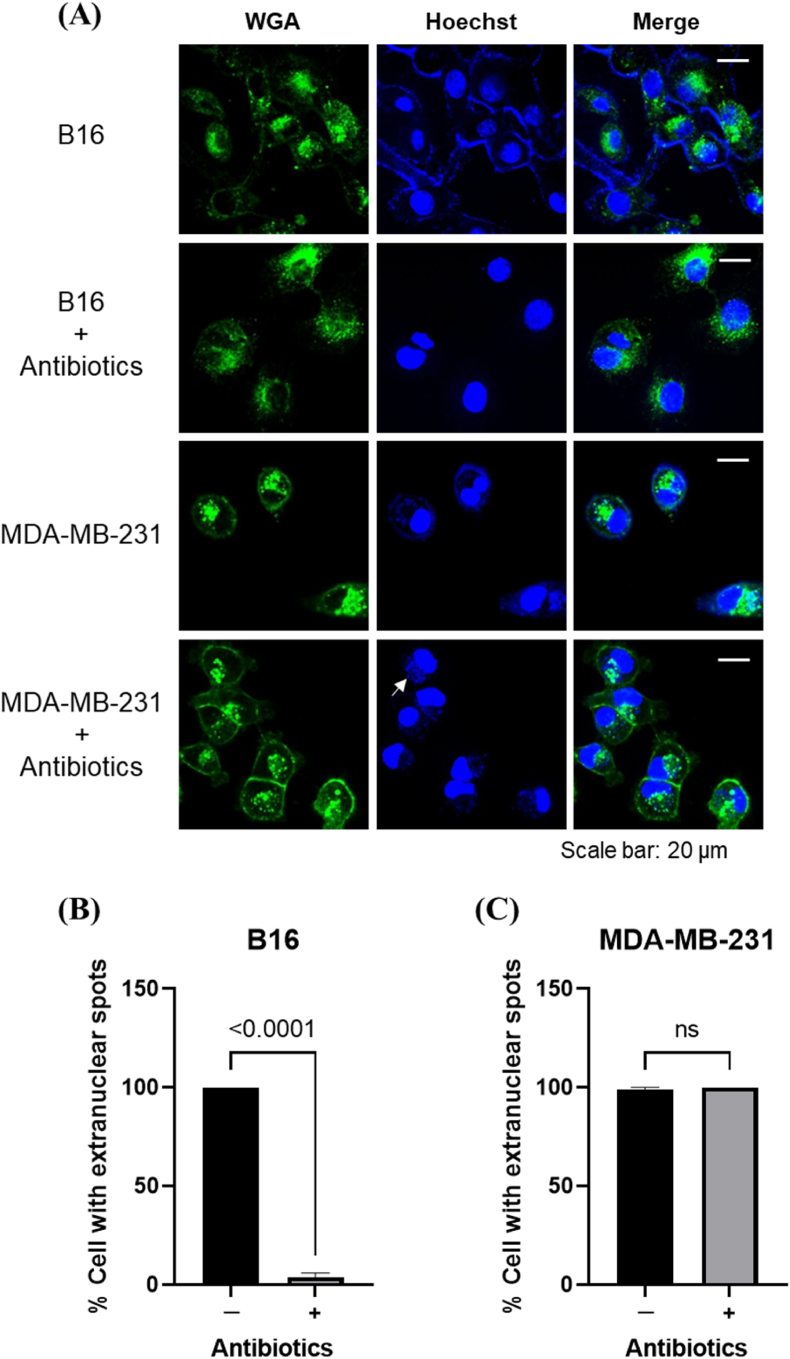
DNA (Hoechst) and plasma membrane (wheat germ agglutinin; WGA) staining mycoplasma-contaminated B16 and MDA-MB-231 cells. **(A)** Confocal micrographs of DNA and plasma membrane-stained cells with or without antibiotic treatment. The proportion of cells with extranuclear spots detected using confocal microscopy (*n* = 8 images) to determine the degree of mycoplasma elimination in antibiotic-treated B16 **(B)** and MDA-MB-231 **(C)** cells, respectively.

After the contaminated cells were treated with antibiotics, the nucleus and cytomembrane were labeled using Hoechst and WGA, respectively (Fig. 3A). We observed that B16 cells had almost no bright extranuclear spots, whereas almost all MDA-MB-231 cells had extranuclear spots (Fig. 3B and C). Therefore, direct DNA staining on its own is not recommended because it often results in false positives, even for cells that are not contaminated with mycoplasma.

**Fig. 4:**
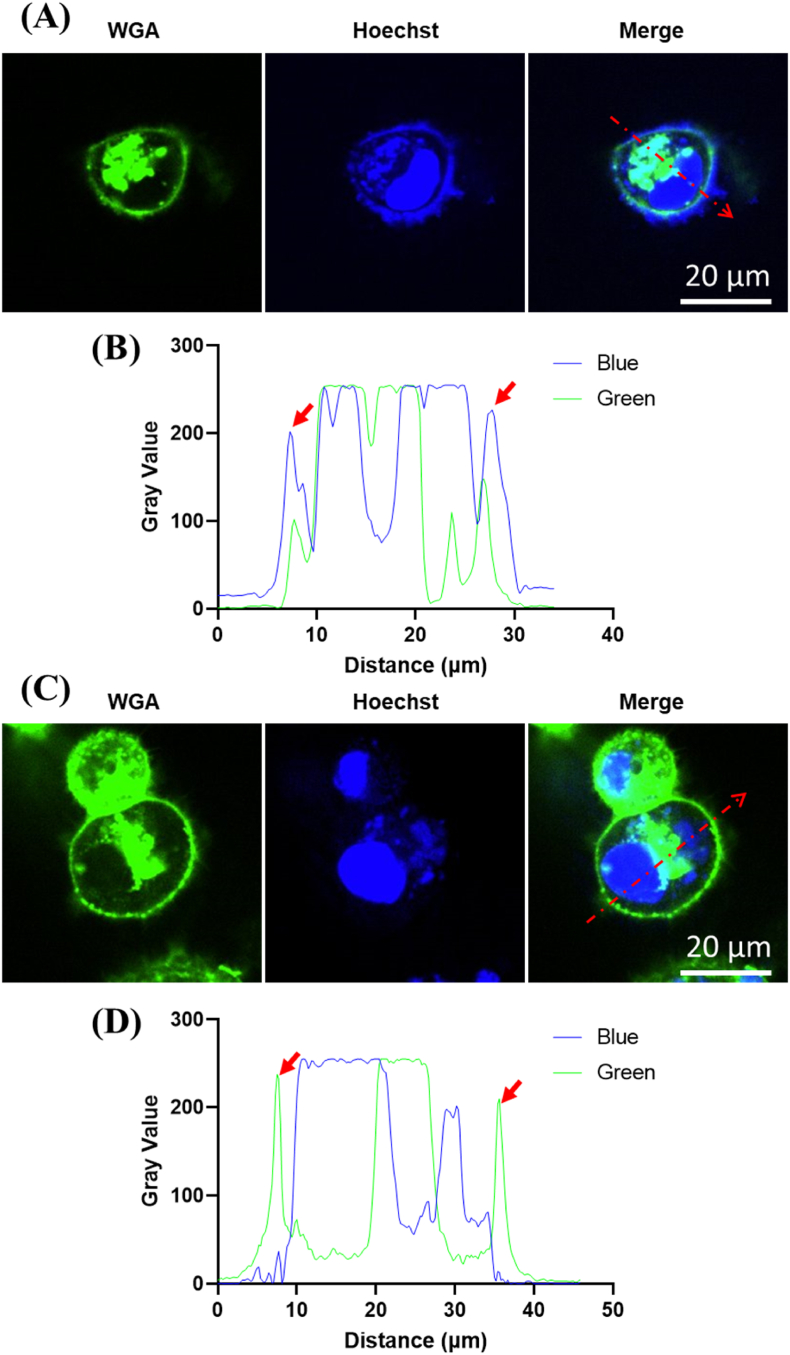
Elimination of interference from cytoplasmic DNA components by colocalization of the cytomembrane and mycoplasma. **(A)** Mycoplasma-contaminated MDA-MB-231 host cells stained with Hoechst (Blue) and WGA (Green); profile line shown by red dotted line. **(B)** Fluorescence gray values of blue and green as a function of distance, with arrows indicating mycoplasma localized to the surface of the host cell membrane. **(C)** Antibiotic-treated MDA-MB-231 host cells stained with Hoechst (Blue) and WGA (Green); profile line indicated by red dotted line. **(D)** Fluorescence gray values of blue and green as a function of distance, with arrows indicating the cell membrane of the host cell.

Since mycoplasma primarily parasitizes the surface of host cells, detecting the colocalization of mycoplasma (Hoechst) with the host cell membrane (WGA) helps avoid false positives caused by cellular DNA in the cytoplasm. In mycoplasma-contaminated MDA-MB-231 host cells, we could clearly see that mycoplasma was localized to the surface of the cell membrane, forming a tight ring, while the cytoplasmic DNA was located inside the ring (Fig. 4A). The fluorescence colocalization of blue and green further illustrated that the peaks (arrows) of mycoplasma were located on the surface of the cell membrane (Fig. 4B) by quantifying the gray value of cell containing either cytoplasmic or membranal spots. However, in antibiotic-treated MDA-MB-231 host cells, the blue fluorescence on the cell membrane surface disappeared (Fig. 4C) and only the peaks (arrows) of the host cell membrane could be seen (Fig. 4D). Although MDA-MB-231 cells with small bright spots in the cytoplasm, confocal micrographs indicated that interference from other DNA components in the cytoplasm (outside the nucleus) was eliminated, allowing the presence of mycoplasma to be easily observed. The colocalization of mycoplasma and the cell membrane yielded results that were consistent with the other detection methods employed (flow cytometry and PCR), representing a great improvement over direct DNA staining. This method is particularly useful when interpretation is challenging, such as in cases where few mycoplasmas are present and cellar DNA may produce small Hoechst fluorescence spots that mimic mycoplasma.

The amount of mitochondrial DNA in a cell is insufficient to cause any interference. Also, although cell debris may be attached to the surface of host cells, it is very unlikely to form tight rings along the plasma membrane. Besides micronuclei, the DNA found in the cytoplasm may result from nuclear fragmentation induced by toxins [12] or apoptosis [13]. The use of Hoechst and WGA fluorescent probes for staining DNA and cell membranes represents an enhanced method for detecting mycoplasma contamination and evaluating its eradication.

This rapid, simple live-cell staining technique is easily applicable and avoids artifacts caused by nuclear fragmentation due to improper handling. By combining Hoechst and WGA staining, mycoplasma and their host cells can be correlated, eliminating interference from cytoplasmic DNA components of the host cell and significantly enhancing the accuracy of DNA staining based on their colocalization.

## CRediT authorship contribution statement

**Mengyuan Li:** Writing – review & editing, Writing – original draft, Visualization, Validation, Supervision, Project administration, Methodology, Investigation, Formal analysis, Data curation, Conceptualization. **Shan Liu:** Investigation, Formal analysis, Data curation. **Cheng Zhou:** Investigation, Data curation. **Mengsi Sun:** Investigation, Data curation. **Yanjie Wang:** Investigation, Data curation.

## Data availability

Data will be made available on request.

## Funding sources

This research did not receive any specific grant from funding agencies in the public, commercial, or not-for-profit sectors.

## Declaration of competing interest

The authors declare that they have no known competing financial interests or personal relationships that could have appeared to influence the work reported in this paper.
